# Effects of improved on-farm crop storage on DNA methylation of mothers and their infants: evidence from a randomized controlled trial in Kenya

**DOI:** 10.1186/s13148-024-01693-z

**Published:** 2024-07-08

**Authors:** Heike Eichenauer, Susanne Fischer, Elena Gardini, Simon Onsongo, Ulrike Ehlert

**Affiliations:** 1 Institute of Psychology, Clinical Psychology and Psychotherapy, University of Zurich, Binzmuehlestrasse 14/Box 26, 8050 Zurich, Switzerland; 2 Aga Khan Hospital, Kisumu, Kenya

**Keywords:** Food insecurity, Pregnancy, DNA methylation, RCT, HPA axis

## Abstract

**Background:**

Stress during pregnancy can lead to adverse maternal and infant health outcomes through epigenetic changes in the hypothalamic–pituitary–adrenal axis. Among farmers in low-income countries, one important stressor is food insecurity, which can be reduced using hermetic storage bags. This study aimed to determine, for the first time, whether a hermetic storage bag intervention during pregnancy positively affects maternal and infant DNA methylation of the hypothalamic–pituitary–adrenal axis-related genes *FKBP5* and *NR3C1*. We further analyzed whether anthropometrics, stress, and mental health were associated with DNA methylation.

**Methods:**

This study was part of a larger matched-pair randomized controlled trial focusing on the impact of improved on-farm storage on food security, poverty, and net income of smallholder farming households. A total of *N* = 149 mothers were recruited by telephone and invited to attend a study appointment at health facilities in Kakamega County, Western Kenya, with their infants in April or May 2021. During the appointment, anthropometric measurements were taken, questionnaires on stress and mental health were administered, and saliva samples were collected. Logistic and multiple linear regression were used to examine the effect of the intervention and related measures on DNA methylation.

**Results:**

Mothers in the intervention group showed higher mean *NR3C1* methylation levels than those in the control group, corrected for multiple testing. Maternal postpartum body mass index was positively associated with infant *NR3C1* CpG3 DNA methylation. The more stressful life events a mother had experienced in the previous 12 months (including during pregnancy), the lower her *FKBP5* CpG3 methylation levels.

**Conclusions:**

Food insecurity and stressful life events during pregnancy seem to exert significant effects on maternal DNA methylation. While these stressors did not appear to impact infant DNA methylation in the present study, maternal postpartum body mass index was significantly related to infant methylation. These findings suggest that while infants may be protected from excessive maternal glucocorticoids by placental barrier activity, maternal metabolic status is still reflected in their epigenetic make-up.

*Trial registration* This study was part of a larger matched-pair randomized controlled trial on the impact of improved on-farm crop storage on welfare, nutrition, and human health. Registration can be found in the American Economic Association (AEA) RCT Registry, RCT ID: AEARCTR-0005845.

## Background

Stress during pregnancy is one of the most important risk factors for poor maternal and infant health, and increased prenatal stress has been associated with pregnancy complications such as miscarriage [1]. Stress during the peripartum is associated with mental disorders, including depression and anxiety in pregnant women [2–4]. In infants, prenatal maternal stress appears to contribute to small for gestational age, preterm birth, and low birth weight [5]. Maternal stress during pregnancy is also a risk factor for deficits in the offspring’s physical, cognitive, and emotional development and related negative health outcomes in adulthood [6–8]. In view of these findings, it is vital to learn more about sources of stress during pregnancy and how they might be reduced to improve maternal and infant health.

The causes of stress during pregnancy are manifold and vary depending on the context. For instance, work-related and relationship-related stress are currently among the most common stressors in high-income countries [9], whereas the causes of stress in low- and middle-income countries are often environmental. One of the most important stressors is food insecurity, defined as inadequate access to food (for physical, social, or economic reasons) required for a healthy life [10]. The prevalence of moderate and severe food insecurity in Africa continues to increase every year, with an estimated one in four people being severely food insecure in 2022 [11]. Our own meta-analytic work on the effects of extreme states of food insecurity and hunger, as occurring during famines, provided clear evidence of significantly increased levels of mental health problems in the aftermath of such events [12].

During pregnancy, exposure to stress such as food insecurity can lead to adverse health outcomes due to alterations in the maternal stress response system. One of the most important stress response systems is the hypothalamic–pituitary–adrenal (HPA) axis with its end product, the glucocorticoid cortisol, with elevated cortisol levels (i.e., hypercortisolism) frequently observed in the initial stages of chronic stress [13] and in stress-related disorders such as depression [14]. Moreover, prenatal stress can also lead to alterations at the epigenetic level [15]. Research interest in this regard has mostly been directed toward the study of deoxyribonucleic acid (DNA) methylation, which describes the addition of methyl molecules to the 5'-carbon position of cytosines, followed by a guanine, to the promoter region of specific genes such as FKBP prolyl isomerase 5 (*FKBP5*). This gene encodes the FK506 binding protein 5, a well-known co-chaperone of glucocorticoid signaling. Hypermethylation of its promoter region is associated with lower gene expression and reduced glucocorticoid sensitivity [16]. Another important gene affecting HPA axis functioning is nuclear receptor subfamily 3 group C member 1 (*NR3C1)*, which encodes the glucocorticoid receptor, with hypermethylation of its promoter region having been linked to lower gene expression and a lower number of glucocorticoid receptors [17]. Research findings indicate that adverse psychopathology potentially induces DNA methylation changes [18–20]. Furthermore, recent studies in people living in low-income countries have provided initial evidence that stressors such as those related to military conflicts and war are associated with altered maternal and infant methylation of *FKBP5* [21, 22] and *NR3C1* [21–23]. However, it is not yet known whether similar effects can be induced by food insecurity.

As stated above, food insecurity in Africa is high, especially in Sub-Saharan Africa (SSA), and this is partly due to post-harvest losses, that is, crops lost after harvesting due to improper storage [24–26]. Fortunately, however, post-harvest losses can be minimized through agricultural measures such as hermetic storage technology [27], with data from randomized controlled trials showing that the administration of hermetic storage bags improves food security for smallholder households [28, 29] and also affects the perceived stress levels and perceived coping abilities of pregnant women from these households [30]. However, to date, no research has examined whether such interventions affect epigenetic changes in HPA axis genes in mothers and their offspring, and how these epigenetic changes relate to maternal mental health and infants’ birth outcomes and growth.

The main objective of the present study was therefore to investigate, for the first time, whether an intervention targeting food insecurity might alter the methylation levels of specific sites within *FKBP5* and *NR3C1* in pregnant women residing in a low-income SSA country. Based on previous research, we hypothesized that: (1a) mothers in the intervention group and 1b) their infants would show higher levels of *FKBP5* methylation and lower levels of *NR3C1* promoter methylation as compared to the control group; (2a) mothers with a more abnormal body mass index (BMI) and (2b) their infants would show lower *FKBP5* methylation levels and higher *NR3C1* methylation levels; (2c) infants with a lower (birth) weight and height would show lower *FKBP5* methylation levels and higher *NR3C1* methylation levels; and (3a) mothers with more stressful life events in the past year, higher levels of perceived stress, worse perceived coping, and more depressive symptoms and (3b) their infants would show comparatively lower *FKBP5* methylation levels and higher *NR3C1* methylation levels.

## Methods

This study was part of a larger matched-pair randomized controlled trial (RCT) on the impact of improved on-farm crop storage on welfare, nutrition, and human health, with a focus on outcomes such as food security, poverty, and net income [31]. The present study was approved by the Kenyatta National Hospital – University of Nairobi (KNH – UoN) Ethics and Research Committee (Approval Number P354/07/2020) and the Kenyan National Commission for Science, Technology, and Innovation (NACOSTI) (License No: NACOSTI/P/21/9673) and conducted in accordance with the tenets of the Declaration of Helsinki.

## Research design of the larger matched-pair randomized controlled trial

The larger matched-pair RCT recruited *N* = 5444 smallholder farmer households in Kakamega County in western Kenya, where maize is the staple crop [28]. This region is affected by a major lean season beginning around April or May, during which food insecurity is high [28]. The intervention was implemented in September 2019, prior to the 2020 lean season. Farmer households randomly assigned to the intervention group received five AgroZ brand hermetic storage bags each, as well as training on how to use them. The storage bags have been shown to successfully minimize post-harvest losses in this population, thereby increasing food security [28, 29]. The control group mostly used traditional polypropylene bags to store their maize. To assess food security status, the farmers received monthly SMS-based mobile phone surveys throughout the intervention period [28]. The surveys included a question asking whether anyone in the household was currently pregnant, and farmers who replied "yes" to this question received an additional survey asking the pregnant women in these households about their approximate due month, perceived stress, and coping (see Eichenauer et al. [30] for more details). Initially, we planned to include all pregnant women expecting to give birth between January and March 2021 in the study. However, as several of these mothers were found to be ineligible (see Additional file 1: Table S1 for reasons), we extended the time period to between December 2020 and April 2021. This is in line with the empirical literature, which suggests that food scarcity during conception and the first trimester has significant effects on DNA methylation [32–34].

### Participants of the present study

From the original sample, *n* = 1093 mothers indicated that they were due to give birth between December 2020 and April 2021. These women were randomly contacted by telephone, in collaboration with staff from the Aga Khan Hospital Kisumu (AKHK) and asked whether they would be interested in participating in the present study. During this telephone call, the mothers were informed about our study and asked about the eligibility criteria. Inclusion criteria were written informed consent to participate and age ≥ 18 years, and exclusion criteria were multiple pregnancy, diseases or treatments that could affect ovarian function before, during, or after pregnancy, use of hormonal supplements, psychotropic medication in the three months prior to the study, drug use and/or smoking, and alcohol consumption of more than one standard unit per day. For infants, maternal written informed consent was obtained before inclusion in the study. The exclusion criteria for the infants were any overt infection, congenital disease, malformation and/or birth injury, and low birth weight (< 1500 g). The final sample consisted of *n* = 149 mothers and *n* = 149 infants. Of these, *n* = 75 mothers and *n* = 75 infants were allocated to the intervention group and *n* = 74 mothers and *n* = 74 infants to the control group.

### Study procedures

Following successful recruitment, health data collection took place from early April 2021 to late May 2021, at various centrally located, easily accessible health facilities within Kakamega County. During data collection, saliva samples were collected by trained researchers from the University of Zurich. The anthropometric data of mothers and infants was collected using weighing scales and tape measures. The questionnaires were completed through an interview, which was carried out either in English or Kiswahili (national language). The AKHK research team was trained in taking anthropometric measurements and in collecting data through telephone and questionnaire-based interviews.

### Measures

#### Sociodemographic and medical data

All sociodemographic information was collected by a questionnaire. Medical (mostly gynecological) history, including birth weight, was obtained from each mother's maternal and child health booklet, which is part of the Kenyan government's public health program for pregnant women (https://www.mchhandbook.com/book/kenya/).

#### Anthropometric data

Infant weight was assessed using a digital electronic infant weighing scale (M112600, ADE Germany GmbH, Hamburg, Germany), and infant height was assessed using a baby height mat (seca 210, seca gmbh & co, Hamburg, Germany).

#### Psychosocial data

Stressful life events experienced in the past year were assessed using questions from the Life Experiences Survey originally developed by Sarason et al. [35] and the Stressful Life Events Questionnaire developed by Cutrona et al. [36], which were adapted to be appropriate for this study population. Perceived stress and coping were assessed using the 4-item version of the Perceived Stress Scale (PSS-4) [37], which has been used previously in the sample population of the present study [30]. The Edinburgh Postnatal Depression Scale (EPDS) was used to determine depressive symptoms during pregnancy and the postpartum period [38, 39]. The stressful life events questions and the EPDS were translated into Kiswahili and back-translated into English by trained bilingual experts. The translation was discussed with our local researchers from AKHK to obtain the most appropriate translation, considering potential cultural differences in different regions of Kenya. Adaptations were subsequently made to ensure that the questions were appropriate to the cultural context of the mothers in our study (see Additional file 1: Table S2 and S3 for the final questionnaires).

#### Biological data

Saliva samples were collected in Oragene kits according to the manufacturer's instructions (OG-500 and OC-175; DNA Genotek, Ottawa, Ontario, Canada) and stored at room temperature until biochemical analysis.

### Data processing and analyses

#### DNA methylation

First, DNA isolation was performed with the PrepIT-L2P protocol (OG-500 and OC-175, DNA Genotek, Ottawa, Ontario, Canada). Next, 500 ng of DNA was used for sodium bisulfite conversion using the EZ DNA Methylation Kit (Zymo Research, Irvine, California, USA). The two target sequences (see Additional file 1: Figures S1 and S2) were amplified using previously published primers for *FKBP5*: frw ACA CTG ACG ACA TGG TTC TAC A NNN GGA TTT GTA GTT GGG ATA ATA ATT TGG and rws 5 TAC GGT AGC AGA GAC TTG GTC T NNN TCT TAC CTC CAA CAC TAC TAC TAA AA and for *NR3C1*: frw 5^′^-TTG AAG TTT TTT TAG AGG G-3^′^ and rws 5^′^-AAT TTC TCC AAT TTC TTT TCT C-3^′^. Universal primer sequences CS1/CS2 at 5' were used for forward and reverse primers (Fluidigm, San Francisco, California, USA). The following polymerase chain reaction (PCR) protocols were used: for *NR3C1* 95 °C for 3 min, then 40x (98 °C for 20 s, 60 °C for 15 s, 72 °C for 15 s), and elongation at 72 °C for 45 s; for *FKBP5* 95 °C for 3 min, then 40x (98 °C for 20 s, 58 °C for 15 s, 72 °C for 15 s), and elongation at 72 °C for 40 s. E-gel size selection was used to purify the obtained amplicons (Thermo Fisher Scientific, Waltham, Massachusetts, USA). A second PCR (95 °C for 3 min, then 10x (98 °C for 20 s, 60 °C for 15 s, 72 °C for 15 s), and elongation at 72 °C for 45 s) was then performed for the purpose of barcoding (Fluidigm, San Francisco, California, USA). The indexed amplicons were pooled, purified, and diluted to 2 nM before sequencing on an Illumina Miseq (Illumina, San Diego, California, USA). Low-quality products were identified and removed (http://www.usadellab.org/cms/index.php?page=trimmomatic) using Trimmomatic v0.35 [40]. The Bismark program (v0.19.0) was used to extract the number of methylated (cytosine) and non-methylated (thymine) bases and only samples with a total count of at least 100 were retained [41]. No sample had to be excluded for *FKBP5* as the coverage was greater than 1600 × for all samples. Thirty infant samples and two maternal samples had to be excluded for *NR3C1*.

#### FKBP5

Based on previous research [19], after DNA methylation laboratory analysis, data points with a still significant deviation (± 3 times the interquartile range (IQR)) were excluded. Some of the data were still skewed after excluding outliers, and the log transformation did not achieve normal distribution; therefore, the Box-Cox transformation [42] was used to approximate normal distribution. Methylation analyses on *FKBP5* were performed using individual cytosine-guanine dinucleotides (CpG) sites (CpG1 to CpG5) and the overall mean methylation of these five CpG sites. Age and BMI have been associated with *FKBP5* DNA methylation [43]. Our analyses showed no significant correlation of maternal age and BMI with *FKBP5* DNA methylation. As monthly household income has been associated with DNA methylation alterations [44, 45] and was significantly associated with CpG sites in our maternal sample, it was included as a control variable for all analyses in mothers. Based on previous research [46–49], infant gestational age and sex were used for analyses in the offspring.

#### NR3C1

Four participants were excluded due to extreme DNA methylation levels (above 30%). We followed the procedure of a previous study by Fiacco et al. [50], who compared individual values and excluded values above 30%, based on information from a review by Palma-Gudiel et al. [51] which indicated that CpG methylation of *NR3C1* is on average mostly below 5%. As the data were still skewed after outliers were excluded, a Box-Cox transformation [42] was performed. Five CpG sites within the NGFI-A binding regions of *NR3C1* exon 1F (CpG2 to 3 and CpG8 to 10) and the overall mean of these five sites were examined [52]. The majority of the sample exhibited *NR3C1* methylation of 0%. This is consistent with Efstathopoulos et al. [53], who also found a high number of unmethylated CpG sites (0%) and therefore categorized participants into unmethylated and methylated groups. Maternal and infant control variables were the same as for *FKBP5*.

#### Statistical analyses

All statistical analyses were performed using R, version 2023.06.1 + 524 [54]. To compare the intervention and control group regarding sociodemographic and outcome variables, Mann–Whitney U tests, Pearson's chi-square (χ2) tests, and Fisher's exact tests were used. Correlation analyses (Spearman or Pearson, depending on normal distribution) were performed to examine the associations between anthropometric variables, psychosocial variables, and DNA methylation. Significant results for outcome variables were further analyzed by performing stepwise linear regression analyses with control variables entered in a first step and predictors entered in a second step. In line with the study by Efstathopoulos et al. [53], for all analyses with *NR3C1*, we applied a two-step approach to analyze our data in order to adequately account for the distribution of available values. First, logistic regressions were calculated to test whether mother and infant could be predicted to have any methylation at all on the gene (0% vs. > 0%). Second, linear regressions were performed with values greater than 0%. Confounding variables were identified by correlation analyses, Pearson’s chi-square test, and Fisher’s exact test. Methylation analyses were adjusted for multiple testing within each gene and its corresponding CpGs (*FKBP5*, *NR3C1*) and within samples (mothers, infants) using the Benjamini–Hochberg method [55] with a false discovery rate of alpha 0.10 based on previous studies [56–58].

## Results

### Participant characteristics

The characteristics of mothers and infants in the intervention and control group are shown in Table 1. There were no statistically significant differences between the intervention and control group regarding sociodemographic, anthropometric, and psychosocial variables, with the exception of infant weight, which was higher in the intervention group compared to the control group (*p* = 0.04); however, infants in the intervention group were also slightly (not significantly) older. Additional file 1: Tables S4 and S5 present the descriptive statistics of all methylation parameters for mothers and infants in the intervention and control group for *FKBP5* and *NR3C1* (including and excluding 0% methylation).

**Table 1:** Sample characteristics of mothers and their infants

	Total		Intervention group		Control group		
	*N (%)*	*M* ± *SD*	*N (%)*	*M* ± *SD*	*N (%)*	*M* ± *SD*	*p*-value
**Mothers**							
Age	149	25.9 ± 6.4	75	26.1 ± 6.3	74	25.7 ± 6.5	ns
Marital status	149		75		74		ns
Married	107 (71.8)		53 (70.7)		54 (73.0)		
Single	38 (25.5)		19 (25.3)		19 (25.7)		
Widowed	1 (0.7)		1 (1.3)		0 (0.0)		
Separated	3 (2.0)		2 (2.7)		1 (1.3)		
Education	149		75		74		ns
None	4 (2.7)		1 (1.3)		3 (4.1)		
Primary	64 (43.0)		37 (49.3)		27 (36.5)		
Secondary	61 (40.9)		26 (34.7)		35 (47.3)		
Tertiary	20 (13.4)		11 (14.7)		9 (12.1)		
Monthly income ($)	148	50	74	56	74	43	ns
BMI	149	23.8 ± 3.8	75	24.0 ± 4.3	74	23.6 ± 3.3	ns
Stressful life events	149	5.1 ± 3.1	75	5.2 ± 2.9	74	5 ± 3.4	ns
PSS-4 Coping	145	6.4 ± 1.7	73	6.4 ± 1.6	72	6.4 ± 1.8	ns
PSS-4 Stress	146	6.2 ± 1.9	75	6.2 ± 1.9	71	6.1 ± 1.9	ns
EPDS	148	13.1 ± 5.5	75	12.9 ± 5.4	73	13.3 ± 5.6	ns
**Infants**							
Age (weeks)	149	10.8 ± 6.0	75	11.3 ± 5.7	74	10.4 ± 6.2	ns
Sex	149		75		74		ns
Boy	81 (54.4)		46 (61.3)		35 (47.3)		
Girl	68 (45.6)		29 (38.7)		39 (52.7)		
BW (kg)	147	3.3 ± 0.5	73	3.2 ± 0.6	74	3.4 ± 0.5	ns
Weight (kg)	148	5.8 ± 1.3	74	6.0 ± 1.3	73	5.6 ± 1.3	0.04
Height (cm)	149	58.1 ± 4.9	75	58.6 ± 4.6	74	57.6 ± 5.1	ns

Stressful life events score ranges from 0 to 19; PSS-4 score ranges from 2 to 10; *p* < .05

*BMI* body mass index, *BW* birth weight, *EPDS* Edinburgh Postnatal Depression Scale,* M* mean, *N* sample size, *ns* not significant, *PSS-4* Perceived stress scale 4-item version, *SD* standard deviation

In the following sections on the effects of the intervention on DNA methylation and on the association between anthropometric measures, maternal stress/mental health and DNA methylation, all multiple regression analyses for mothers were adjusted for monthly income, and for infants for gestational age and sex of the infant.

### Effects of the intervention on DNA methylation

#### Mothers

The intervention did not significantly predict mean *FKBP5* methylation or methylation of any of the individual CpG sites (all *p* > 0.10).

Regarding *NR3C1*, logistic regressions revealed that mothers in the intervention group were significantly more likely to have high levels of *NR3C1* CpG3 methylation than mothers in the control group (OR = 2.17, 95% CI 1.11, 4.32). In multiple linear regressions (see also Table 2), the intervention showed a significant effect on *NR3C1* CpG3 methylation and on overall mean *NR3C1* methylation. After controlling for multiple testing, the intervention effect on CpG3 vanished, while the effect on overall mean *NR3C1* methylation remained significant. With a mean monthly income, the overall mean *NR3C1* methylation was 0.23% in mothers in the control group and 0.43% in mothers in the intervention group.

**Table 2:** Effect of the intervention on *FKBP5* and *NR3C1* methylation

	**Mothers**		**Infants**	
	adjusted *b*	*p*-value	adjusted *b*	*p*-value
***FKBP5***				
CpG1	− 1.36E + 23*	0.277	222,677*	0.052
CpG2	− 2.90E + 15*	0.105	0.970^a^	0.587
CpG3	− 503.400*	0.518	− 0.010	0.052
CpG4	− 2.661^a^	0.046	0.369	0.458
CpG5	− 1.439^a^	0.316	0.400	0.391
Mean	− 0.839^a^	0.261	− 0.004	0.091
***NR3C1***				
CpG2	0.051	0.067	− 0.006	0.939
CpG3	0.060	0.027	0.897^b^	0.325
CpG8	0.133^b^	0.699	0.010^b^	0.989
CpG9	− 0.043^b^	0.914	0.118	0.541
CpG10	− 0.283^b^	0.484	0.102	0.465
Mean	0.057	**0.011**	0.000	0.995

Box-Cox transformed beta values. Maternal analyses are adjusted for monthly income and infant analyses are adjusted for gestational age and sex of infant. *p* < .05 (value in bold remained significant after adjustment for multiple testing). * CpG* cytosine-guanine dinucleotides, *FKBP5* FKBP prolyl isomerase 5, *NR3C1* nuclear receptor subfamily 3 group C member 1, mean NGFI-A binding regions of the *NR3C1* exon 1F (mean of CpG2-3 and CpG8-10) and mean of *FKBP5* CpG1 to 5

^*^The Box-Cox transformed adjusted b values are high because the non-transformed distribution is very skewed to the right and therefore has a high lambda value (see Additional file 1: Figure S3)

^a^No transformation, original values were normally distributed

^b^Log-transformed values as lambda was 0

#### Infants

The intervention did not significantly predict mean *FKBP5* and *NR3C1* methylation or methylation of any of the individual CpG sites (all *p* > 0.05). For more detailed information, see Table 2.

### Association between anthropometric measures and DNA methylation

#### Maternal BMI and maternal DNA methylation

Maternal BMI did not predict overall mean *FKBP5* and *NR3C1* methylation or methylation of any of the individual CpG sites (all *p* > 0.05).

#### Maternal BMI and infant DNA methylation

Maternal BMI did not predict mean *FKBP5* methylation or methylation of any of the individual CpG sites (all *p* > 0.05).

**Fig. 1:**
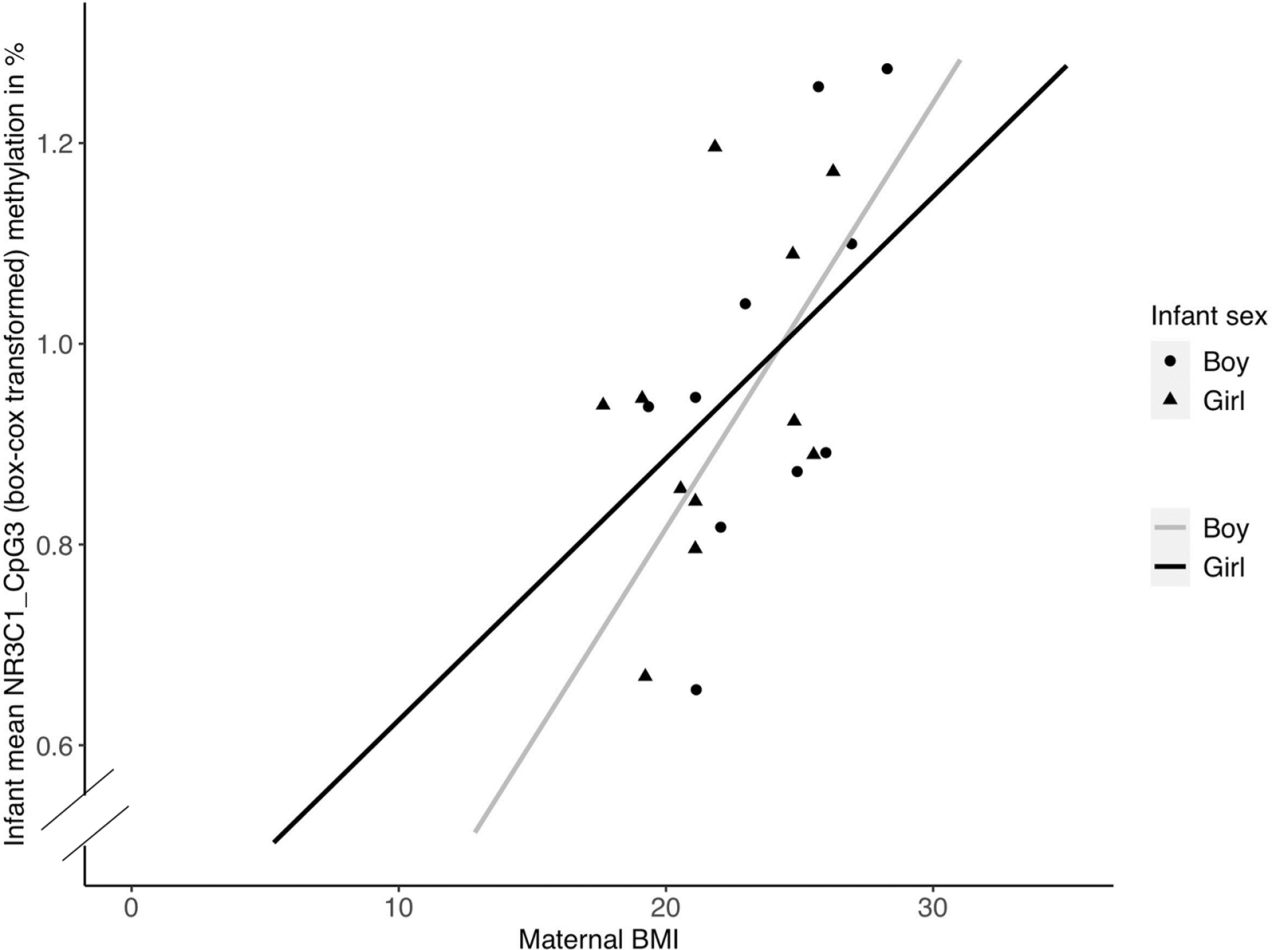
Scatter plot showing the association between maternal BMI and methylation of *NR3C1* CpG3 (Box-Cox transformed) in infants separately by infant sex. Black and gray lines represent regression lines

Regarding *NR3C1*, logistic regression analyses revealed that maternal BMI was not a significant predictor of DNA methylation (all *p* > 0.05). In multiple regression analyses, the examination of individual CpG sites revealed a significant effect of maternal BMI on infant DNA methylation at CpG3 (*ß* = 0.038, *t*(15) = 3.081, *p* = 0.008; see Fig. 1). This association remained significant after correction for multiple testing. Maternal BMI accounted for 33% of the variance in CpG3 methylation. Female infants with an average gestational age and with a mother with an average BMI at the time of data collection had a DNA methylation of CpG3 of 0.90%, compared to 0.80% for male infants.

#### Infant (birth) weight/height and infant DNA methylation

Infant anthropometric outcomes did not predict mean *FKBP5* and *NR3C1* methylation or methylation of any of the individual CpG sites (all *p* > 0.05).

### Association between maternal stress/mental health and DNA methylation

#### Mothers

**Fig. 2:**
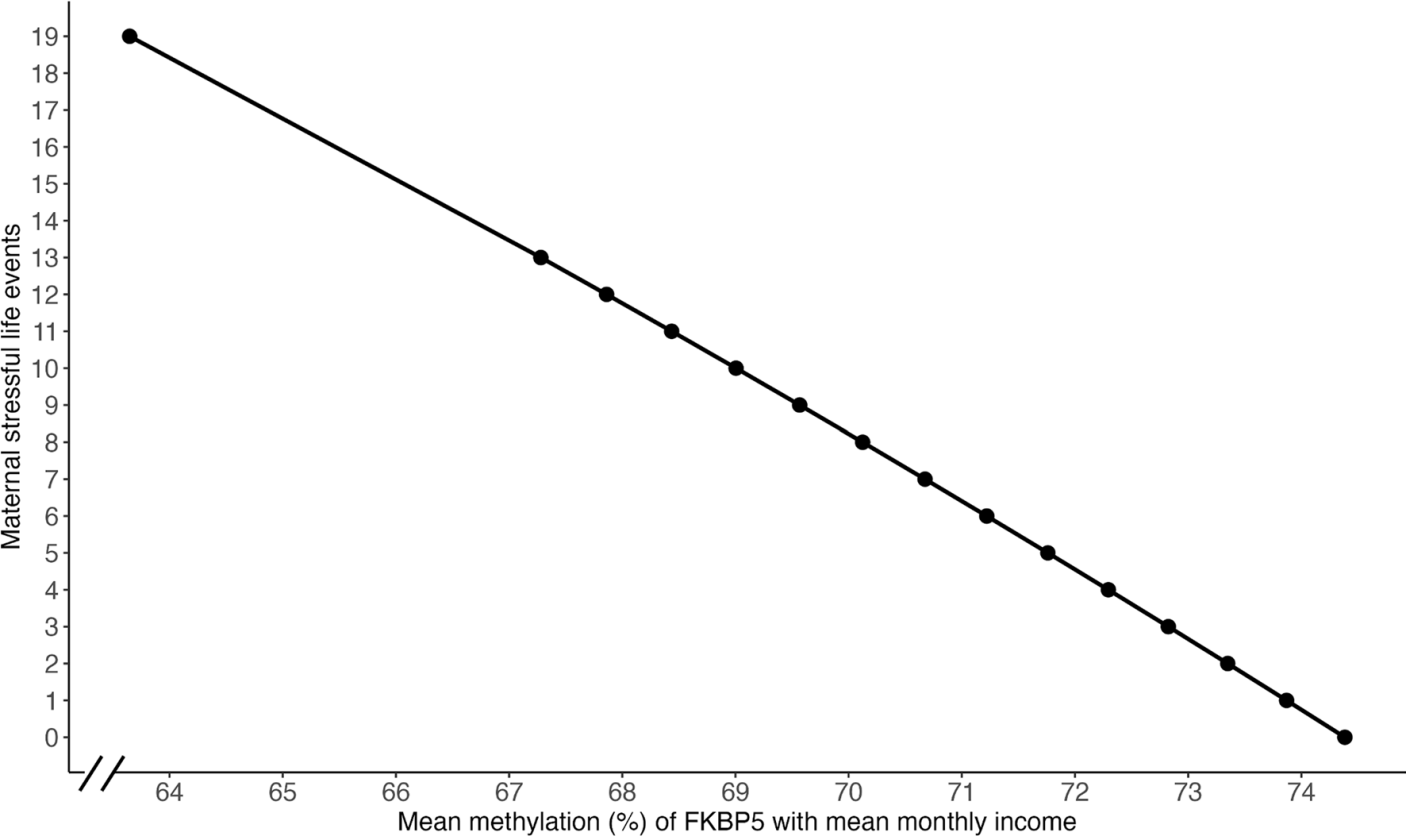
Association of maternal stressful life events (in the case of an average monthly income) in the past 12 months and maternal *FKBP5* CpG3 methylation. The sample size was 144 mothers. One mother had experienced 19 stressful life events in the past year at the time of data collection. Removing this participant did not change the results (see Additional file 1: Table S6)

Mothers who experienced more stressful life events in the past year were significantly more likely to have lower *FKBP5* CpG3 methylation as compared to mothers with fewer stressful life events in the past year (*ß* = -319.6, *t*(142) = -2.621, *p* = 0.01; see Fig. 2). Stressful life events accounted for 6% of the variance in CpG3 methylation. This finding remained significant after correcting for multiple testing. No other effects of maternal stress or mental health on maternal *FKBP5* methylation were detected (all *p* > 0.05).

Regarding *NR3C1*, neither logistic regression nor multiple linear regression analyses revealed maternal stress and mental health to be significant predictors of DNA methylation (*p* > 0.05).

#### Infants

Neither logistic regression nor multiple linear regression analyses revealed maternal stress and mental health to be significant predictors of mean *FKBP5* and *NR3C1* methylation or methylation of any of the individual CpG sites (all *p* > 0.05).

## Discussion

In the present study, we investigated whether a food security intervention in pregnant women living in Kenya was associated with altered maternal and infant DNA methylation levels of specific sites within *FKBP5* and *NR3C1*. We further analyzed whether anthropometric measures, maternal stress, and mental health were linked to changes in *FKBP5* and *NR3C1* DNA methylation. Three main findings emerged: First, mothers in the intervention group had increased mean *NR3C1* methylation compared to mothers in the control group. Second, maternal BMI was a significant predictor of infant *NR3C1* CpG3 methylation, insofar as the higher a mother's BMI, the higher her infant's *NR3C1* CpG3 methylation. Third, the more stressful life events a mother had experienced in the past 12 months (including during pregnancy), the lower her *FKBP5* CpG3 methylation.

Research indicates that CpG sites within exon 1F of *NR3C1* are predominantly hypermethylated following stress exposure [51], suggesting that an intervention that reduces a stressor, such as food insecurity, should result in lower methylation within *NR3C1*. Contrary to expectation, our study revealed increased *NR3C1* methylation in mothers in the intervention households compared to those in the control households. However, our findings are consistent with Eichenauer et al. [30], who also reported increased perceived stress in pregnant women from the intervention households. To explain this finding, the authors suggested that in line with Lazarus and Folkman's transactional stress theory [59, 60], the improved on-farm storage technology may have elicited feelings of uncertainty, uncontrollability, and threat due to the farmers’ lack of experience in using these bags, thus triggering a stress response. As mentioned previously, hypermethylation of *NR3C1* is associated with lower gene expression [17], meaning that fewer glucocorticoid receptors are available. Since suppression of HPA axis activity occurs via a negative feedback mechanism [61], our finding suggests a hypercortisolemic pattern in the mothers [62], which did not transfer on to their infants.

In addition to stress exposure, unfavorable maternal body composition adversely affects maternal and offspring health, suggesting an involvement of epigenetic changes [63–65]. We hypothesized that in a food-insecure region, mothers would tend to under-consume food because they are more likely to give it to their children [66], and would therefore have a lower BMI. Thus, a higher (healthier) maternal BMI would have positive effects on maternal and infant DNA methylation (hypermethylation of *FKBP5* and hypomethylation of *NR3C1*). Although we found no significant association between maternal BMI and maternal DNA methylation, higher maternal postpartum BMI was linked to increased methylation of *NR3C1* CpG3 in the offspring, although this finding needs to be interpreted with caution as the effect size was small. A one-unit increase in maternal BMI was associated with a 0.038% increase in methylation. In addition, as mentioned above, *NR3C1* methylation is difficult to detect, and due to our two-step approach, only 21 infants had *NR3C1* CpG3 methylation levels above 0%. A further reason for the null findings and weak association may be that regardless of whether participants were in the intervention or control households, maternal BMI was within the range of the WHO BMI criteria for normal weight [67], and evidence only indicates a change in DNA methylation of *FKBP5* and *NR3C1* with a BMI below or above the WHO criteria [64, 68–70].

Not only is the prenatal period the time when gene-environment interactions are most pronounced and can lead to permanent epigenetic changes in glucocorticoid signaling, but (cumulative) stressors across the lifespan also have the potential to induce DNA methylation changes in adulthood [71]. Indeed, our results showed a negative association between the number of stressful life events a mother experienced during pregnancy and her *FKBP5* CpG3 methylation. The finding of reduced *FKBP5* methylation due to adverse stressors supports a previous review by Matosin et al. [16] and suggests increased gene expression [62] and an increased risk for the development of mental disorders [16]. By contrast, stressful life events were not associated with maternal *NR3C1* methylation in the present study, which might be explained by the fact that *NR3C1* methylation is more resistant to change and thus more stable in comparison with *FKBP5* [72]. Indeed, changes methylation within intron 7 of *FKBP5* have been detected even after short stress management training or psychotherapy, whereas *NR3C1* does not appear malleable to such interventions [73–75].

The present study is the first to examine the effects of improved on-farm storage technology on maternal and infant DNA methylation. As a further strength, the study adds to the growing literature on DNA methylation, stress, and mental health. However, a limitation lies in the lack of pre-intervention (baseline) DNA methylation data, meaning that we cannot rule out pre-existing differences between the intervention and control group, although the randomization and relatively large sample size renders this unlikely. Moreover, maternal BMI was measured postpartum, and no pre-pregnancy BMI was available. An issue not addressed in this study was whether differences in the ethnicity of the study population may have affected our methylation results. However, a descriptive statistic from 2013/2014 indicated that 90% of the household heads in Kakamega County were from the Luhya ethnic group [76], suggesting that most participants were also from this largest ethnic group present in Kakamega County. It can therefore be assumed that if only a small number of participants were from a different ethnic group, this would probably not have had a strong impact on the analyses. Furthermore, to our knowledge, research on significant differences in DNA methylation based on ethnicity has tended to focus on more diverse ethnic groups, e.g., Caucasian vs. Asian populations (for review, see [77–79]). In addition, a study by Galanter et al. [80] reported that within an ethnicity, although belonging to a different ethnic subgroup accounted for over 60% of differentially methylated loci, environmental and socioeconomic factors contributed to over 30% of the differential methylation patterns. Another limitation is that the tissue in which the epigenetic changes were measured may have influenced the results. For example, in a study by Armstrong et al. [81], the authors reported that analyses of DNA methylation changes within 7 gene loci (including *NR3C1*) in placental tissue, cord blood and saliva did not correlate significantly with each other, suggesting that these tissues cannot be substituted for each other. This indicates that DNA methylation changes are tissue specific*.* Future studies should also focus on epigenome-wide studies, as environmental, psychological, and physiological stressors can induce epigenetic changes throughout the genome [82]. Most importantly, a follow-up study should examine whether the altered DNA methylation pattern for *NR3C1* persists over time. Since the time of data collection, mothers may have experienced the benefits of the intervention through a significant reduction in food insecurity after the first harvest season [28], potentially altering their appraisal of the intervention as a "threat" [30] and resulting in a reduced stress response and thus more favorable methylation patterns.

## Conclusions

The increased perceived stress due to the use of hermetic storage bags reported in our previous study [30] is also reflected in a dysregulation of the HPA axis due to increased *NR3C1* methylation in the mothers. Additionally, our results support previous findings on the involvement of the gene *FKBP5*, which regulates glucocorticoid signaling, in the response to stressful life events during pregnancy. Furthermore, the dysregulation of the maternal HPA axis due to the improved on-farm storage technology as well as the maternal stressful life events during pregnancy did not significantly affect the infants’ DNA methylation. This suggests that they may have been protected from excessive maternal glucocorticoid exposure by the activity of the placental barrier [83]. However, maternal BMI during the postpartum period was associated with increased *NR3C1* methylation in the infant, suggesting that maternal metabolic status was still reflected in their epigenetic make-up.

## Data Availability

The datasets used and/or analyzed during the current study are available from the corresponding author on reasonable request.

## Abbreviations

AKHK: Aga Khan Hospital Kisumu
CpG: Cytosine guanine dinucleotides
DNA: Deoxyribonucleic acid
EPDS: Edinburgh Postnatal Depression Scale
FKBP5: FKBP prolyl isomerase 5
HPA: Hypothalamic-pituitary-adrenal
IQR: Interquartile range
KNH-UoN: Kenyatta National Hospital—University of Nairobi
NACOSTI: Kenyan National Commission for Science, Technology, and Innovation
NR3C1: Nuclear receptor subfamily 3 group C member 1
PCR: Polymerase chain reaction
PSS-4: Perceived Stress Scale, 4-item version
RCT: Randomized controlled trial
SSA: Sub-Saharan Africa
